# Efficient Calculation of the Negative Thermal Expansion in ZrW_2_O_8_

**DOI:** 10.3389/fchem.2018.00296

**Published:** 2018-07-30

**Authors:** Fernando D. Vila, Scott T. Hayashi, John J. Rehr

**Affiliations:** Department of Physics, University of Washington, Seattle, WA, United States

**Keywords:** zirconium tungstate, NTE, DFT, quasi-harmonic approximation, phonon density of states

## Abstract

We present a study of the origin of the negative thermal expansion (NTE) on ZrW_2_O_8_ by combining an efficient approach for computing the dynamical matrix with the Lanczos algorithm for generating the phonon density of states in the quasi-harmonic approximation. The simulations show that the NTE arises primarily from the motion of the O-sublattice, and in particular, from the transverse motion of the O atoms in the W–O and W–O–Zr bonds. In the low frequency range these combine to keep the WO_4_ tetrahedra rigid and induce internal distortions in the ZrO_6_ octahedra. The force constants associated with these distortions become stronger with expansion, resulting in negative Grüneisen parameters and NTE from the low frequency modes that dominate the positive contributions from the high frequency modes. This leads us to propose an anharmonic, two-frequency Einstein model that quantitatively captures the NTE behavior.

## 1. Introduction

There has been considerable interest in recent years in developing materials with electronic and structural properties tuned toward specific applications. Likewise, there is broad interest in pre-screening potential materials using theoretical simulations in an effort to minimize their complexity and simplify their synthesis. Although great progress has been made in the prediction of many properties (Jain et al., 2013) such as band gaps, electronic properties, equilibrium structures, and spectra, thermal properties depend on additional calculations of vibrational properties and often remain difficult for first principles computational methods. This is typically due to the complexity of the materials under consideration. For example, complex ceramics like ZrW_2_O_8_ with large unit cells have heretofore been too demanding for routine screening using first principles calculations of both electronic and vibrational properties with conventional electronic structure codes. On the other hand, efficient calculations of many vibrational properties are now possible. For example, we have previously shown (Poiarkova and Rehr, 2001; Krappe and Rossner, 2002; Vila et al., 2007) that an efficient Lanczos algorithm for the projected phonon density of states (PDOS) obtained from standard density functional theory (DFT) calculations (Lee and Gonze, 1995; Rignanese et al., 1996; Baroni et al., 2001) of the dynamical matrix (DM) and the quasi-harmonic approximation (Allen and De Wette, 1969; Boyer, 1979) can produce accurate Debye-Waller factors for EXAFS and x-ray crystallography.

Here we develop an extension of this approach for efficient calculations of the thermal properties of ZrW_2_O_8_, a quintessential example of negative thermal expansion (NTE) ceramics, in which the NTE is large over a broad range of temperatures.

The origin of the NTE in ZrW_2_O_8_ is controversial, having been previously attributed to a variety of mechanisms. For instance, the Rigid Unit Mode (RUM) model (Hammonds et al., 1996; Pryde et al., 1996, 1997; Hancock et al., 2004; Tucker et al., 2005, 2007) suggests that the tetrahedra and octahedra that make up the structure are mostly unaffected by the active modes. In contrast, other works (Cao et al., 2002, 2003; Bridges et al., 2014) have suggested that these units are distorted while the Zr-W distance remains more or less unchanged. Yet another alternative (Mary et al., 1996; Gupta et al., 2013; Sanson, 2014) suggests that the Zr–O–W bonds bend, pulling the Zr and W atoms closer and thus causing the NTE. In this paper we take advantage of the local nature of the PDOS to study the origin of the NTE. This procedure allows us to partition the contributions to the Helmholtz free energy from different atomic sites and deduce the origin of NTE from the shifts in the local free energy minima. In a second study in this collection (Vila et al., under review) we apply these methods to study the effects of the NTE on the EXAFS properties of the system, such as the EXAFS mean square relative displacements for relevant single- and multiple-scattering paths (Cao et al., 2003), as well as the anisotropic behavior of the crystallographic mean square atomic displacements. The following sections present a brief summary of the Lanczos algorithm for the PDOS, show how we compute the dynamical matrix efficiently, give a detailed discussion of the volume and temperature effects on the force constants, potentials and PDOS, and present a simple two-frequency model that reproduces the observed NTE quite accurately.

## 2. Methods

### 2.1. Helmholtz free energy

The quasi-harmonic approximation (QHA) (Allen and De Wette, 1969; Boyer, 1979) has been widely used to study thermal effects on the structural properties of both crystalline (Erba et al., 2015) and molecular solids (Erba et al., 2016; Brandenburg et al., 2017). QHA simulations usually rely on the full diagonalization of the force constant matrix obtained with density functional perturbation theory (DFPT) or other analytic derivative approaches (Baroni et al., 2010; Erba, 2014). We have previously shown (Vila et al., 2007) that it can also be combined with the Lanczos algorithm in order to avoid the full diagonalization and efficiently compute many thermal properties of materials. Briefly, the lattice thermal expansion *a*(*T*) can be obtained by minimizing the Helmholtz free energy *A*(*a, T*) at a given *T*,

(1) $$\begin{array}{l} \frac{\partial A(a,T)}{\partial a}{|}_{a=a(T)}=0. \end{array}$$

Within the quasi-harmonic approximation *A*(*a, T*) is given by

(2) $$\begin{array}{l} A\left(a,T\right)=U\left(a\right)+F\left(a,T\right), \end{array}$$

where *U*(*a*) is the internal energy of the electronic system in its ground state, and *F*(*a, T*) is the total vibrational free energy (VFE) of the system

in the Born-Oppenheimer approximation. For convenience we define *F*(*a, T*) = *F*_0_(*a*) + *F*_*T*_(*a, T*). Here *F*_0_(*a*) and *F*_*T*_(*a, T*) are the zero-point and temperature-dependent components, respectively. They can be calculated in terms of the density of vibrational modes of the lattice ρ(*a*, ω) at a given *a*,

(3a) $$\begin{array}{l} F_{0}\left(a\right)=\frac{\hbar }{2}\overset{\infty }{\underset{0}{\int }}\omega \;\rho \left(a,\omega \right)d\omega , \end{array}$$

(3b) $$\begin{array}{l} F_{T}\left(a,T\right)=k_{B}T\overset{\infty }{\underset{0}{\int }}\ln\;\left[1-e^{-\hbar \omega /k_{B}T}\right]\;\rho \left(a,\omega \right)d\omega , \end{array}$$

where

(4) $$\begin{array}{l} \rho \left(a,\omega \right)=\underset{i\alpha }{\sum }\rho_{i\alpha }\left(a,\omega \right) \end{array}$$

is the total PDOS, and ρ_*iα*_(*a*, ω) is the PDOS projected onto the α = {*x, y, z*} coordinate of atom *i*. For simplicity we assume in this work that the system contracts isotropically with a cubic unit cell of lattice constant *a* and ignore equilibrium distortions.

### 2.2. Projected phonon density of states

Although highly simplified phenomenological approaches like the Einstein and Debye models based on empirical data can be quite useful, they are generally inadequate to treat complex materials (Dimakis and Bunker, 1998; Poiarkova and Rehr, 1999). As an alternative which overcomes many of these limitations Poiarkova and Rehr (1999, 2001) introduced a method for EXAFS calculations in which the unit-normalized PDOS, projected onto a simple bond or arbitrary multiple-scattering path, is calculated from the imaginary part of the lattice dynamical Green's function. Vila et al. (2007) introduced an extension of the method to compute the PDOS projected onto atomic displacements ρ_*iα*_(*a*, ω),

(5) $$\begin{array}{l} \rho_{i\alpha }\left(a,\omega \right)=-\frac{2\omega }{\pi }\text{Im}\langle i\alpha \left|\frac{1}{\omega^{2}-\text{D}\left(a\right)+i\epsilon }\right|i\alpha \rangle . \end{array}$$

Here |*iα* > is a Lanczos seed vector representing a normalized, mass-weighted displacement of atom *i* along the Cartesian direction α such that the position of the center of mass of the cell is not changed, and **D**(*a*) is the *dynamical matrix* (DM) of force constants computed at lattice constant *a*,

(6) $$\begin{array}{l} D_{jl\alpha ,j^{\prime }l^{\prime }\beta }\left(a\right)={\left(M_{j}M_{j^{\prime }}\right)}^{-1/2}\;\frac{\partial^{2}U\left(a\right)}{\partial u_{jl\alpha }\partial u_{j^{\prime }l^{\prime }\beta }}, \end{array}$$

where *u*_*jlα*_ is the displacement of atom *j* in unit cell *l* along the direction α, and *M*_*j*_ is the mass of atom *j*. As described below, **D**(*a*(*T*)) can be interpolated from **D**(*a*), which makes the force constants depend parametrically on the temperature and thus includes the dominant effects of anharmonicity. Efficient calculations of the lattice dynamical Green's function can be accomplished using a continued fraction representation, with parameters obtained with the iterative Lanczos algorithm (Deuflhard and Hohmann, 1995). This approach yields an efficient many-pole representation for the PDOS; since the pole locations can be interpreted as Gaussian quadrature points (Haydock, 1980), the method is well suited for accurate spectral integrations, as needed for example in the calculation of the VFE. The first iteration of the Lanczos algorithm corresponds to a *correlated Einstein* model for the |*iα* > displacement such that ρ_*iα*_(*a*, ω) = δ(ω−ω_*E*_) with an Einstein frequency $\omega_{E}^{2}=\langle i\alpha |\text{D}\left(a\right)|i\alpha \rangle$. Poiarkova et al. showed that a second tier continued fraction gave about 10% errors for EXAFS Debye-Waller factors, while Krappe and Rossner (2002) later showed that six iterations are needed to achieve convergence to within 1%. We have found (Vila et al., 2007) that 16 or more iterations may be needed to achieve the same precision for ρ_*iα*_(*a*, ω). Nevertheless, this algorithm avoids the explicit calculations of phonon-spectra and hence permits highly efficient calculations.

### 2.3. Dynamical matrix

The main computational bottleneck to using this approach for many complex materials of industrial or technological interest then lies in obtaining sufficiently accurate DMs. Although empirical or model potential estimates of the interatomic force constants are sometimes available, they are not useful in screening a broad range of potential new materials. Moreover, their temperature dependence usually limits their accuracy and generality. Nevertheless, we have previously shown (Vila et al., 2007) that *ab initio* DMs obtained from density functional theory (DFT) calculations provide accurate force constants that can be used to compute both EXAFS Debye-Waller factors and thermal expansion. In that study we restricted our attention to simple periodic systems using the methodology implemented in ABINIT (Gonze et al., 2002), described in detail in Gonze and Lee (1997). This method computes ${\tilde{D}}_{j\alpha j^{\prime }\beta }\left(a,\text{q}\right)$, the dynamical matrix in reciprocal space:

(7) $$\begin{array}{l} {\tilde{D}}_{j\alpha j^{\prime }\beta }\left(a,\text{q}\right)=\underset{l^{\prime }}{\sum }D_{j0\alpha ,j^{\prime }l^{\prime }\beta }\left(a\right)e^{i\text{q}\cdot \left({\text{R}}_{j^{\prime }l^{\prime }}-{\text{R}}_{j0}\right)}, \end{array}$$

and then Fourier transforms it back into real space. This approach is efficient for systems with simple unit cells of just a few atoms and high symmetry. In this paper we extend this method by using our recently developed parallelization scheme (Vila et al., Submitted) for the efficient computation of the DM in more complex unit cells based on first principles DFT calculations. This method computes the real space DM in Equation (6) directly using finite differences in supercells, taking advantage of the real space efficiency of codes like VASP (Kresse and Hafner, 1993, 1994; Kresse and Furthmüller, 1996a,b; Kresse and Joubert, 1999). This real space approach is particularly well suited for large unit cells with low symmetry such as those in supercell simulations of supported nanoparticles. In such cases the use of analytic methods such as DFPT results in very demanding calculations both in computing time and memory. A similar approach has recently been described for molecular systems (Liu et al., 2017) that suggest that the high availability of commodity processors can be used to take advantage of the parallelization of the finite differences. Similarly, our approach also relies on the parallel computation of complete rows of the DM, directly from the analytical DFT forces in VASP as a function of the lattice constant:

(8) $$\begin{array}{l} \frac{\partial^{2}U\left(a\right)}{\partial u_{jl\alpha }\partial u_{j^{\prime }l^{\prime }\beta }}=\frac{\partial F_{jl\alpha }\left(a\right)}{\partial u_{j^{\prime }l^{\prime }\beta }}, \end{array}$$

where *F*_*jlα*_ is the force on atom *j* in unit cell *l* along the direction α. For the case of ZrW_2_O_8_ treated here we use centered finite differences with displacements of 0.015 Å, which provide sufficiently accurate force constants. The most efficient row partition of the DM depends on the configuration of the computing system used. Details for the partition used here are presented in section 2.5.

### 2.4. Other considerations

As discussed in section 2.1, the temperature-dependence of the lattice constant *a*(*T*) is obtained by minimizing the free energy *A*(*a, T*) in Equation (2) with respect to *a* at a given temperature *T*. The temperature dependence can be obtained quite efficiently since it only requires the recomputation of the integral in Equation (3) given a PDOS ρ_*iα*_(*a*, ω) at a certain lattice constant. The variation with lattice constant, however, requires the recomputation of ρ_*iα*_(*a*, ω), and thus of **D**(*a*), which is the most computationally demanding step. In previous work (Vila et al., 2007) we solved this issue for simple materials by computing *A*(*a, T*) in a lattice constant grid that can be fitted or interpolated to find the minimum lattice constant as a function of temperature. To improve efficiency we took advantage of two properties of these simple systems: (i) we assumed that the shape of *A*(*a, T*) can be accurately described using a Morse potential form as a function of lattice constant; and (ii) we reduced the number of calculations of **D**(*a*) by taking advantage of the nearly linear behavior of **D**(*a*) as a function of lattice constant near equilibrium,

(9) $$\begin{array}{l} \text{D}\left(a\right)={\text{D}}_{2}+{\text{D}}_{3}\left(a-a_{0}\right) \end{array}$$

where **D**_2_ is the constant harmonic DM at *a*_0_, the lattice constant of minimum internal energy, and **D**_3_ is the constant matrix of anharmonic constants. This parametrization assumes that anharmonic contributions of order higher than cubic are small, and allows us to compute the DM for only two lattice constants that can be used to determine **D**_2_ and **D**_3_. This approach typically cuts the computational time by a factor of about 2/3 or more. Given the complexity of some of the systems in the present study and the importance of having reliable efficiency enhancements for computational screening of materials, here we have opted to verify that this approximation is still valid by computing the DMs over a more complete grid of lattice constants. We have also changed the form used to fit *A*(*a, T*) to a simple polynomial.

### 2.5. Computational details

All structural optimizations and DM calculations were performed with VASP (Kresse and Hafner, 1993, 1994; Kresse and Furthmüller, 1996a,b; Kresse and Joubert, 1999) using PAW potentials (Kresse and Joubert, 1999) and the PBEsol exchange-correlation functional (Perdew et al., 2008). An 8 × 8 × 8 *k*-point grid was used in all calculations, which was sufficient to achieve converged results. To reduce the effect of Pulay stress, the planewave energy cutoff was set at 350 eV. The finite difference force calculations used a three point centered stencil with 0.015 Å displacements. To obtain the variation of the DM with respect to lattice constant, we start by optimizing the size of unit cell and the reduced positions of the atoms in it while preserving its symmetry. This produces an optimized unit cell expanded by 1% from the experimental value. Given that the reduced positions do not change with volume until the phase transition at about 430 K (Evans et al., 1999), we freeze them and expand/contract around the optimal lattice size. We compute the DM at expansions of 0.0, 0.5, 1.0, 1.5, and 2.0% with respect to the experimental lattice constant, which correspond to contractions of 0.5 and 1.0%, and expansions of 0.5 and 1.0% with respect to the optimal lattice contant. This procedure provides an adequate number of points for the interpolation of both the energies and components of the DM. For this material and taking into consideration the computing system being used, we partitioned the DM into 44 independent blocks, one per atom in the simulation cell, each using 512 processors for a total of 22,528. This procedure reduced the computing time from 33 h/core to 0.8 h/core. Equivalent simulations using DFPT in ABINIT would require on the order of 30–40 h/core, with a maximum number of processors of about 200–300.

## 3. Results and discussion

### 3.1. Structure

**Figure 1:**
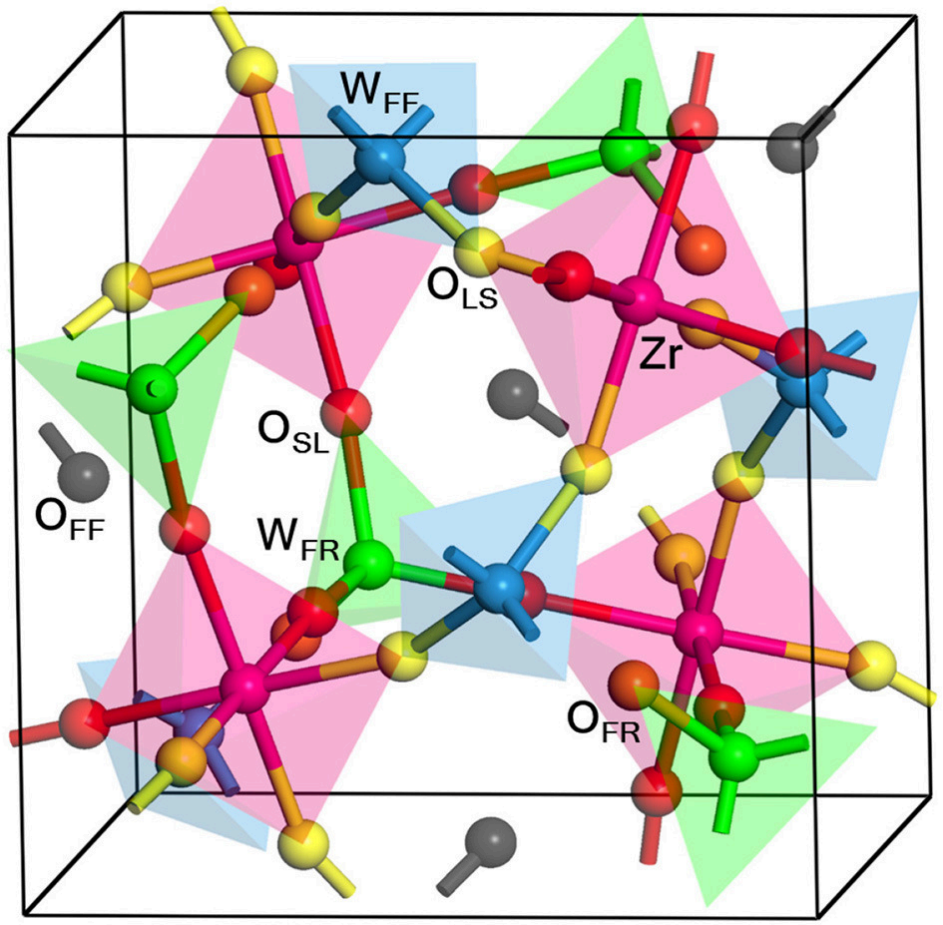
Structure of the ZrW_2_O_8_ cell. The cell is composed of four equivalent Zr atoms (magenta), eight W atoms, of type W_FF_ (blue) and W_FR_ (green), and 32 O atoms of type O_LS_ (yellow), O_SL_ (red), O_FR_ (orange) and O_FF_ (black). Examples of the different types of O and W atoms are highlighted, and the coordination tetrahedra and octahedra are also included for clarity. The W_FF_, W_FR_, O_LS_, O_SL_, O_FR_, and O_FF_ atoms correspond, respectively, to the W_1_, W_2_, O_1_, O_2_, O_3_, and O_4_ in the traditional notation (Mary et al., 1996).

**Figure 2:**
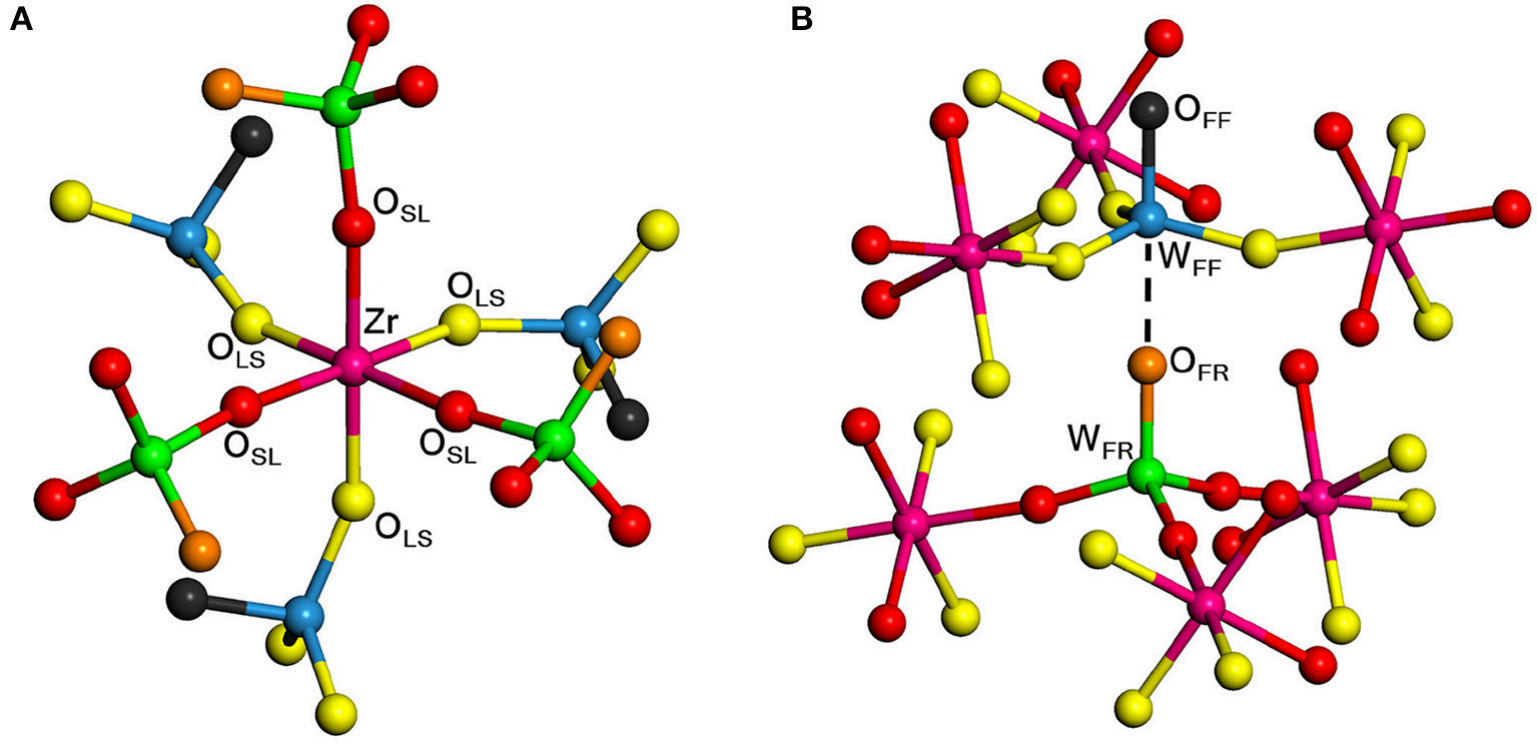
Local structure of ZrW_2_O_8_ around the Zr octahedra **(A)** and W tetrahedra **(B)**. The color key is the same as in Figure 1. Examples of the different types of O and W atoms are highlighted. The bottom figure shows the pairing of the two types of W atoms. The W_FF_, W_FR_, O_LS_, O_SL_, O_FR_, and O_FF_ atoms correspond, respectively, to the W_1_, W_2_, O_1_, O_2_, O_3_, and O_4_ in the traditional notation (Mary et al., 1996).

Figure 1 shows the structure of the unit cell of ZrW_2_O_8_ used here. The cell is composed of four ZrO_6_ octahedra linked to eight WO_4_ tetrahedra. The ZrO_6_ units are all equivalent, with three short and three long Zr–O bonds, each connecting to a WO_4_ unit (Figure 2A). The WO_4_ units have three long W–O bonds, connecting to the ZrO_6_ units, and a short one that is essentially free (Figure 2B). Unlike the ZrO_6_ units, there are two types of WO_4_ units depending on the direction of the free W–O bond: Four of them point toward a ZrO_6_ unit and have a truly free W–O bond, while the other four point to another WO_4_ unit and have a more restricted W–O bond. These two types of tetrahedra are paired (Figure 2B). Here we label the different types of atoms in the cell as follows: O_SL_ and O_LS_ are O atoms bridging WO_4_ and ZrO_6_ units, with short W–O and long Zr–O bonds, and vice versa. They correspond, respectively, to the O_2_ and O_1_ atoms in the more traditional notation (Mary et al., 1996). The O_FF_ and O_FR_ are O atoms in truly free and somewhat restricted W–O bonds, respectively. They correspond to the O_4_ and O_3_ atoms in the traditional notation (Mary et al., 1996). Finally, the W atoms bonded to O_FF_ and O_FR_ center are similarly labeled W_FF_ and W_FR_, and correspond to the W_1_ and W_2_ atoms in the traditional notation (Mary et al., 1996), respectively. Table 1 presents a comparison between the experimental (Auray et al., 1995) and optimized structural parameters obtained in the PBEsol optimization. The changes in internal structure are minimal except for the overall expansion of the cell of 1% predicted by PBEsol.

**Table 1:** Experimental and theoretical structural parameters for the ZrW_2_O_8_ cell used in this work.

**Parameter**		**Expt**.	**PBEsol**
Lattice		9.155	9.246
Bonds	W_FR_–O_FR_	1.736	1.751
	W_FR_–O_SL_	1.785	1.797
	W_FF_–O_FF_	1.712	1.728
	W_FF_–O_LS_	1.799	1.826
	Zr–O_SL_	2.092	2.119
	Zr–O_LS_	2.051	2.062
	Zr–W_FR_	3.867	3.909
	Zr–W_FF_	3.751	3.795
Angles	Zr-O_SL_–W_FR_	171.5	173.2
	Zr-O_LS_–W_FF_	153.9	155.0

*All distances in Å and angles in deg. The experimental values are taken from Auray et al. (1995). See text and Figure 1 for an explanation on the labels*.

### 3.2. Bond force constants

**Figure 3:**
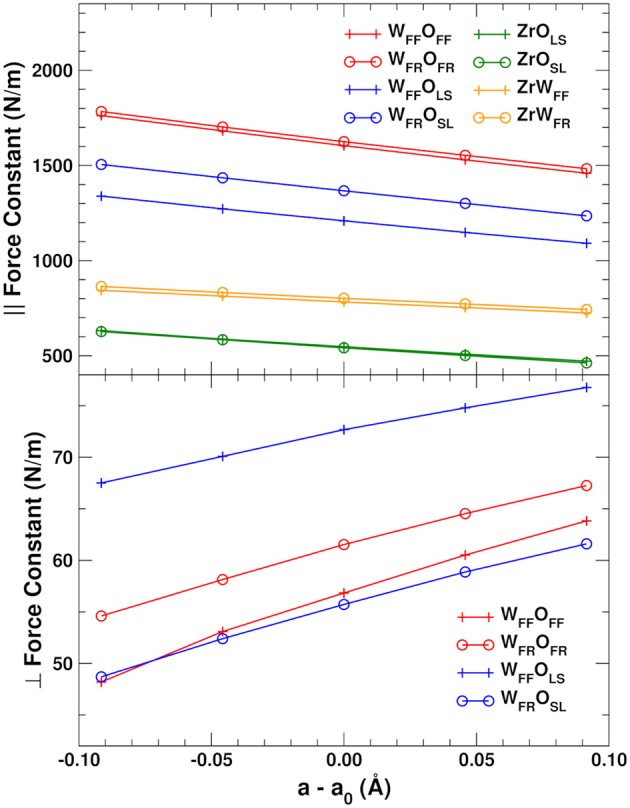
Mean force constants for the parallel OW and OZr stretch **(Top)** and perpendicular OW scissoring **(Bottom)** displacements in ZrW_2_O_8_. The perpendicular force constants are averaged over two directions in the plane orthogonal to the W–O bond for the W_FF_–O_FF_ and W_FR_–O_FR_ bonds, and over the in-plane and out-of-plane directions for the W_FF_–O_LS_ and W_FR_–O_SL_ bonds. See text for further details.

Figure 3 shows the lattice constant dependence of the mean force constants for the parallel stretch and perpendicular scissoring displacements for the different bond types in ZrW_2_O_8_ (The numerical data for this and all other plots in this work can be found in Supplementary Data Sheet 1). The parallel force constants were obtained by rotating the 6 × 6 ${\left(jl,j^{\prime }l^{\prime }\right)}_{\alpha ,\beta }$ blocks of the DM formed by *jl* and *j*′*l*′ near-neighbor atoms into a local coordinate system along the bond. The scissors force constants were obtained similarly by rotating the 3 × 3 (*jl, jl*)_α, β_ block for O atom *jl* into coordinates parallel and perpendicular to the W–O bond. For the W_FF_–O_FF_ and W_FR_–O_FR_ bonds, motion of the O atoms orthogonal to the bonds is nearly equivalent in all directions. For the W_FF_–O_LS_ and W_FR_–O_SL_ bonds, we average over the in-plane and out-of-plane directions defined by the W–O–Zr plane. These directions are also nearly equivalent, with anisotropies of at most 20%.

We find that of all the elements of the DM, only those associated with the O atoms exhibit the positive anharmonicity essential to observe NTE. Indeed, only the deviations perpendicular to the bonds have positive slope (Figure 3, bottom). The force constants associated with the perpendicular O distortions are also much weaker than the parallel ones, and are therefore activated at much lower temperature. The strength of the parallel bond distortions follows closely the trends in the bond distances shown in Table 1, with the shorter W_FF_–O_FF_ and W_FR_–O_FR_ bonds having the largest force constants, followed by the slightly longer W_FF_–O_LS_ and W_FR_–O_SL_ bonds. As discussed in section 2.4, the components of the dynamical matrix should vary mostly linearly with cell expansion in simple systems. Given the large NTE observed for ZrW_2_O_8_, which implies the existence of significant anharmonicity, it is worth examining the accuracy of the linear approximation. Figure 3 shows that the linearity of the bond-parallel force constants is excellent for small displacements, while the transverse ones show a slight curvature. The mean absolute errors from a linear approximation are ± 3 N/m for the strong parallel displacements and only ± 0.4 N/m for the transverse ones. Consequently, the linear approximation can be used to estimate the the lattice dependence of the dynamical matrix to high accuracy.

### 3.3. Helmholtz and vibrational free energies

**Figure 4:**
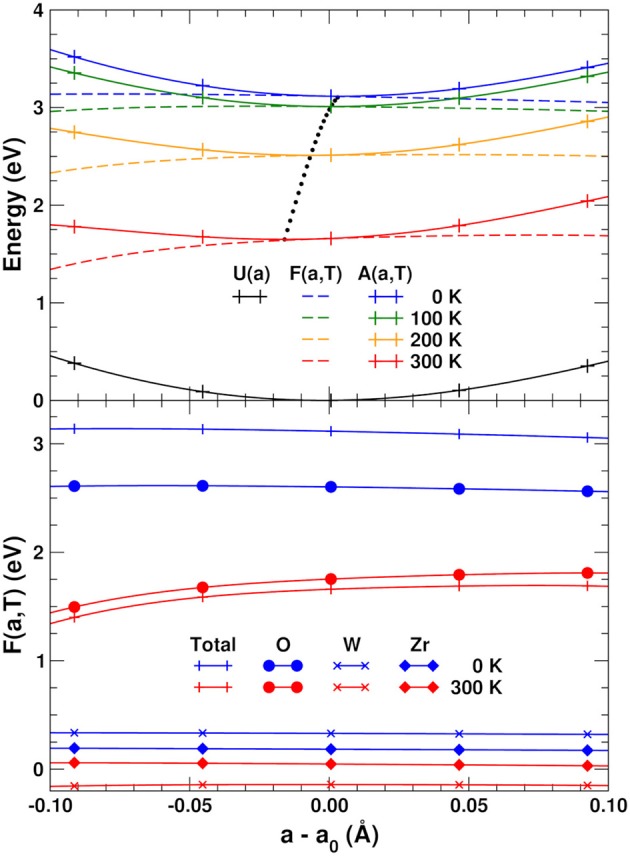
**(Top)** Internal energy *U*(*a*), total vibrational free energy *F*(*a, T*) and Helmholtz free energy *A*(*a, T*) as a function of lattice constant and temperature. The potentials are interpolated using a 5th-order polynomial. The black dots indicate the optimal lattice constant as a function of temperature. **(Bottom)** Total *F*(*a, T*) and contributions from the O, W, and Zr sites.

Figure 4 (top) shows the variation with lattice constant and temperature of the internal energy *U*(*a*), total vibrational free energy *F*(*a, T*), and Helmholtz free energy *A*(*a, T*), together with the optimal lattice constant as a function of temperature obtained by fitting *A*(*a, T*) to a 5th-order polynomial and minimizing numerically. The NTE clearly arises from the lower relative VFE at smaller lattice constants. To help understand the origin of this behavior, the bottom of Figure 4 also shows a decomposition of the VFE at low and high temperature, obtained by partitioning the sum over atoms in Equation (3) into its O, W, and Zr components. This decomposition reveals that the W and Zr contributions to the VFE are virtually constant with variations in lattice constant; consequently the variation of total VFE closely follows the variation of only the O component. This further substantiates our finding that the perpendicular O distortions are the primary origin of NTE in ZrW_2_O_8_.

### 3.4. Negative thermal expansion

**Figure 5:**
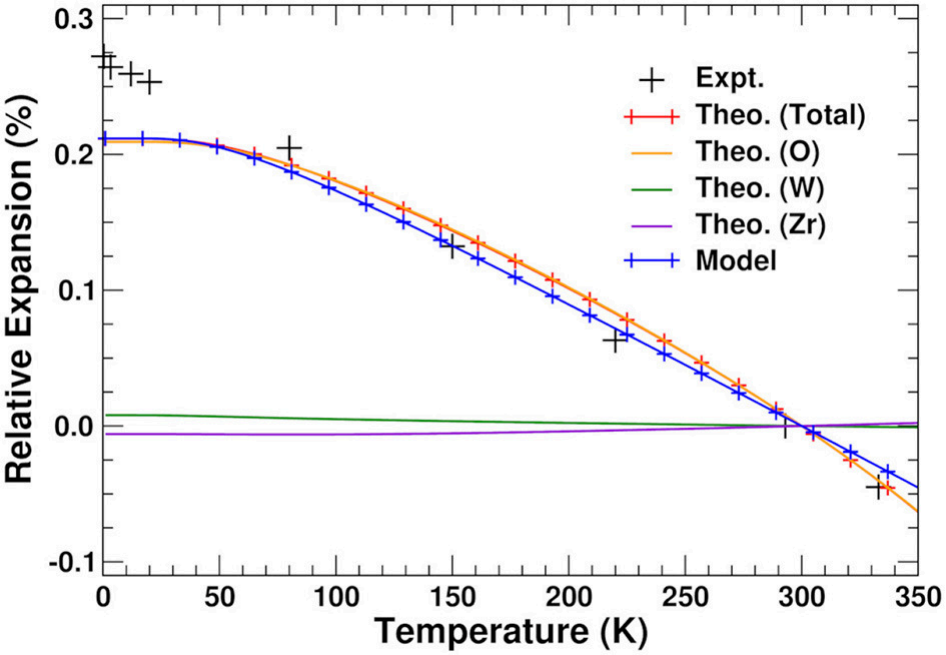
Total, O, W, and Zr components, and results from our two-pole model (see text) of relative thermal expansion in ZrW_2_O_8_ as a function of temperature. The experimental results are taken from Evans et al. (1996).

The relative thermal expansion obtained by minimizing *A*(*a, T*) with respect to lattice constant is shown in Figure 5. The agreement with experiment is clearly very good except at very low temperatures, where the theoretical curve shows the expected flattening due to zero-point effects, which does not appear to be pronounced in the experiment (Evans et al., 1996). Figure 5 also shows the NTE that would be obtained by only considering the components of the VFE arising from the O, W, and Zr lattices. As expected from the previous discussion, the NTE arises exclusively from the O component. The Zr and W contributions have a minimal effect on the system, in contrast with the hypothesis (Mary et al., 1996; Gupta et al., 2013; Sanson, 2014) that the bending of the Zr–O–W bonds causes the NTE by pulling the Zr and W atoms together.

### 3.5. Total and projected phonon densities of states

**Figure 6:**
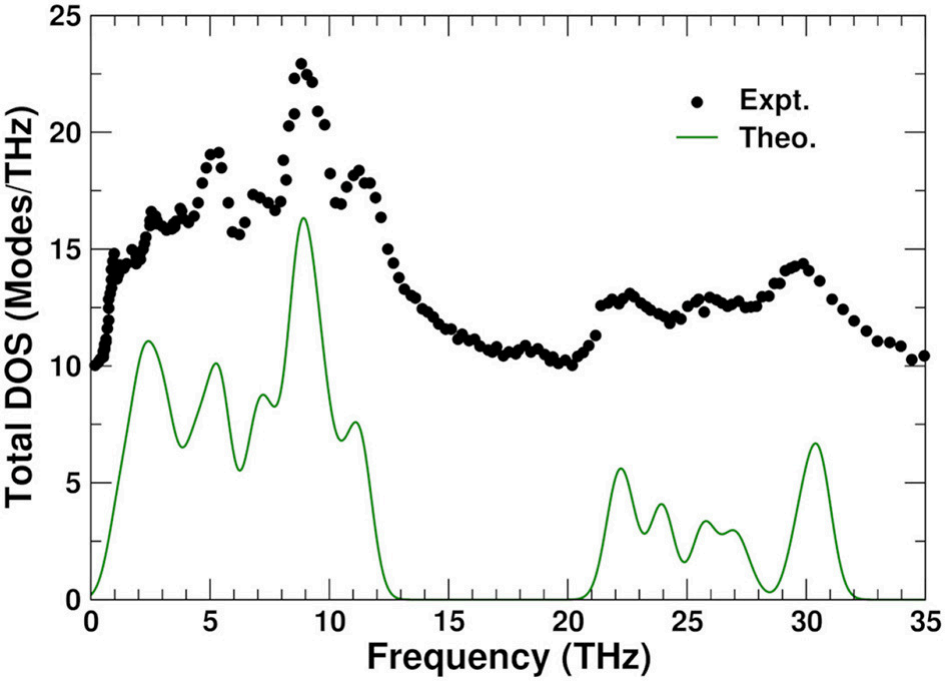
Comparison of the theoretical (0 K) and experimental (Ernst et al., 1998) (300 K) total phonon density of states (DOS) in ZrW_2_O_8_. The experimental result has been vertically shifted for clarity.

Figure 6 shows a comparison between the theoretical total density of states at 0 K and experiment (Ernst et al., 1998) obtained from inelastic neutron scattering at 300 K. The PDOS can be roughly divided into three regions: high frequency range (above 20 THz), associated with bond stretching modes, middle range (between 5 and 13 THz), corresponding mostly to bond bending modes, and low frequency range (below 5 THz), which are thought to correspond to librational and translational modes (Chaplot, 2005). The overall agreement is excellent; the small discrepancy at the lowest frequencies around 1 THz, where the Lanczos approach yields a single peak, is likely due to the size of the 44-atom unit cell used in the calculations.

**Figure 7:**
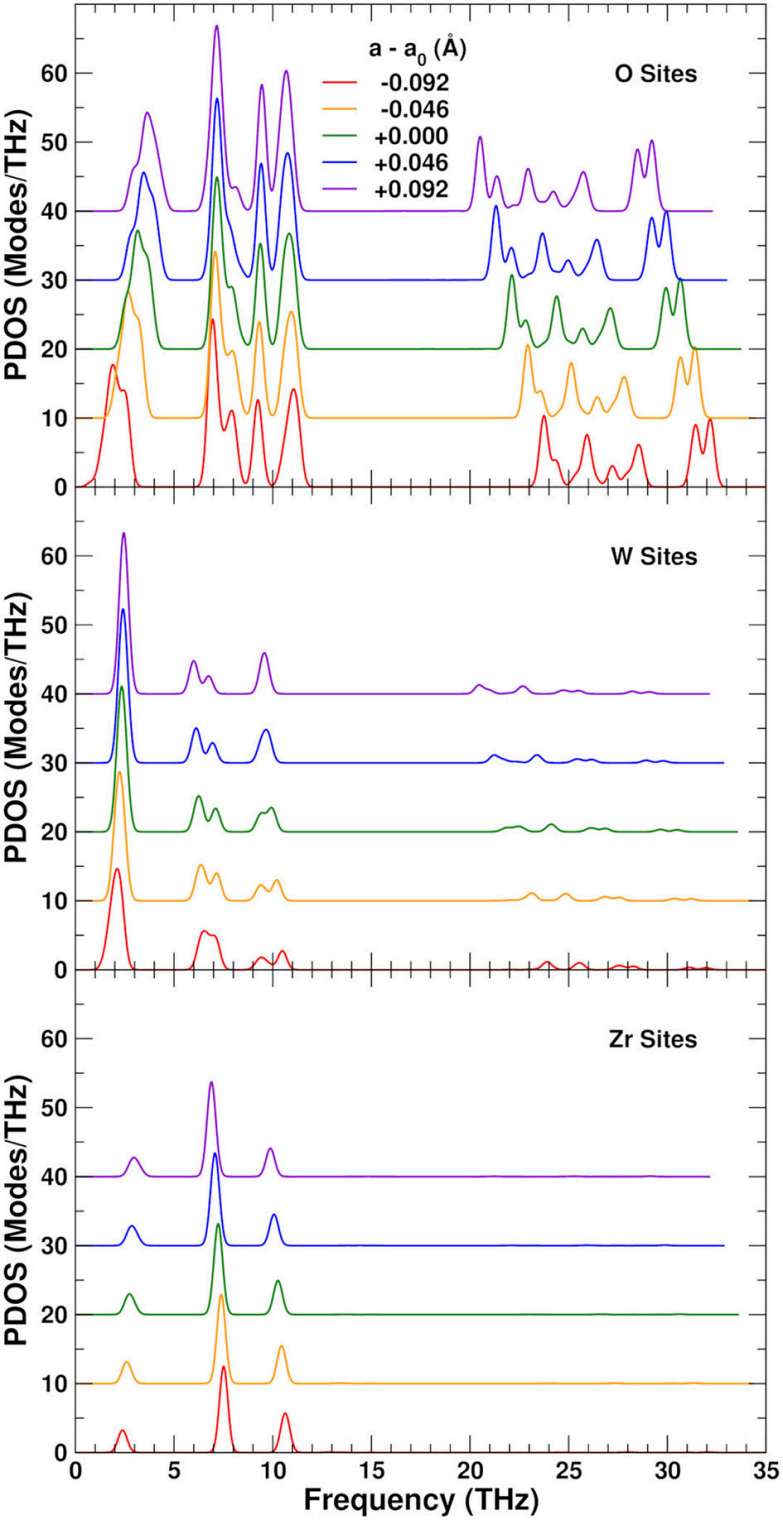
Variation of the projected phonon density of states (PDOS) as a function of lattice constant in ZrW_2_O_8_. The partial PDOS projected onto O, W, and Zr sites are shown in the top, middle and lower panels, respectively. The curves for different lattice constants have been vertically shifted for clarity.

**Figure 8:**
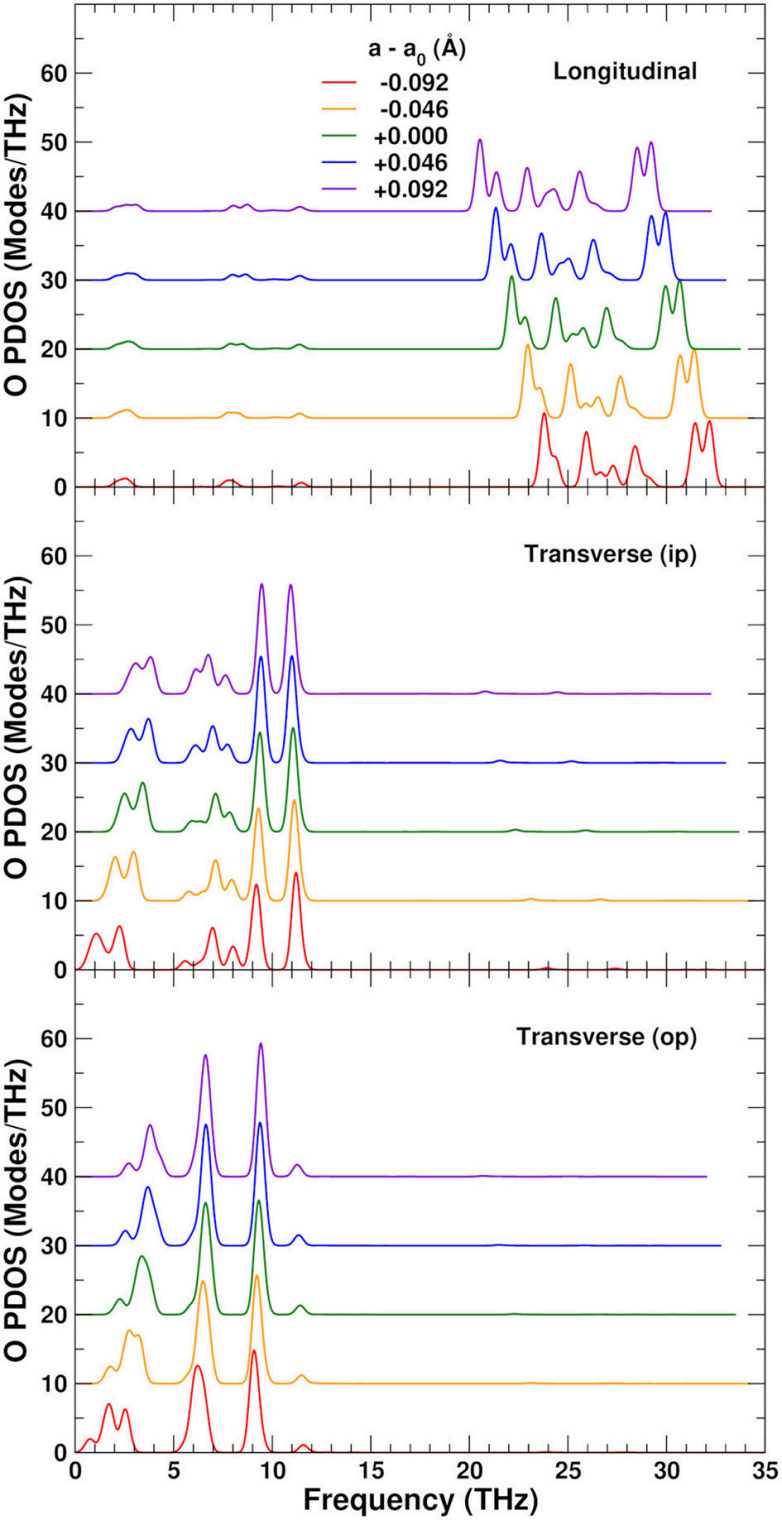
Variation of the O-sites phonon density of states (PDOS) as a function of lattice constant in ZrW_2_O_8_, projected onto longitudinal (along the O–W or Zr–O–W direction), and transverse in-plane (ip) and out of plane (op) displacements. The curves for different lattice constants have been vertically shifted for clarity.

To understand the physical origin of the NTE more completely, we consider the behavior of the projected phonon density of states, or PDOS. Figure 7 shows the PDOS projected onto the O, W, and Zr sites. The high frequency PDOS only has significant weight on the O sites. The W and Zr sites have most of their weight in the low and medium regions, respectively. The variation of the PDOS with lattice constant depends greatly on the projection site as shown by the Grüneisen parameters γ = *d*lnω/*d*ln*V* of the different sites and frequency ranges: The middle range is essentially independent of lattice constant with a γ = − 0.3 ± 0.3, except for the Zr sites which show a very small decrease of the frequencies with expansion and γ = 2.0. This positive γ results in the very small tendency to normal behavior seen in the Zr component of the thermal expansion shown in Figure 5. The high frequency range, found only on the O sites, also contributes to positive thermal expansion with a γ = 1.9 ± 0.2. These high frequencies result from the strong parallel force constants discussed in section 3.2, as demonstrated in Figure 8, which shows that the DOS projected onto the longitudinal (i.e., along the O–W or Zr–O–W direction) motion of the O atoms only has weight in the high frequency range. These stretch modes become weaker as the system expands, and would result in net positive thermal expansion if not countered by the low frequency region. In fact, if the high frequency range is neglected, the relative NTE at 0 K is overestimated by about 30%. The low frequency region exhibits a large negative γ = − 11 ± 2 for the O sites, and about γ = − 3 ± 1 for the W sites, in agreement with our finding that the W sites have a small contribution to the NTE, while the O sites account for most of it. For the overall low frequency range we estimate the same γ of − 8 ± 4, as in previous theoretical estimates, (Gupta et al., 2013) which is in reasonable agreement with experimental estimates ranging from − 7 to − 20 (Hancock et al., 2004). Figure 8 also shows the projection onto the transverse motion of the O atoms, which is localized in the medium and low frequency ranges, as expected for bending and librational modes, respectively.

**Figure 9:**
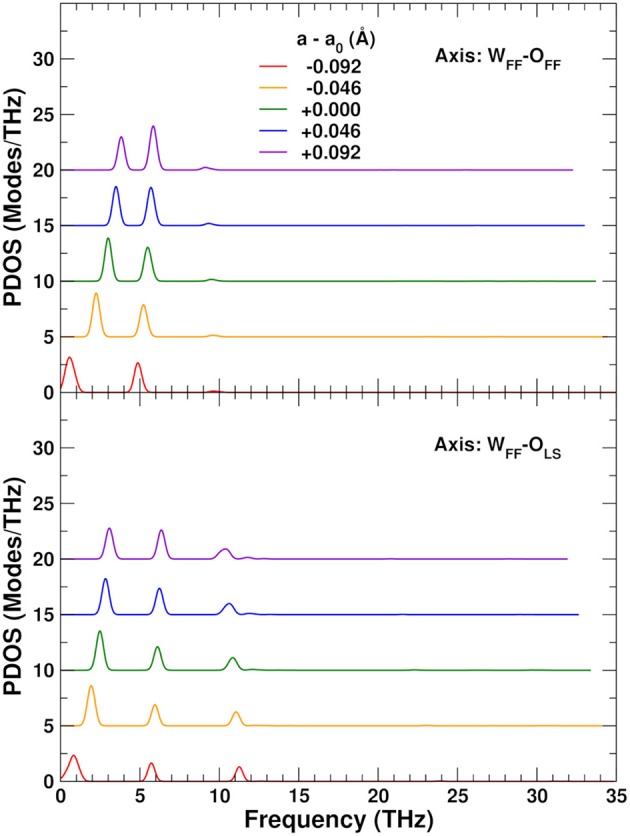
Variation of the ZrW_2_O_8_ phonon density of states (PDOS) projected onto rotational coordinates around the W_FF_–O_FF_ and W_FF_–O_LS_ axes, as a function of the lattice constant. The curves for different lattice constants have been vertically shifted for clarity.

**Figure 10:**
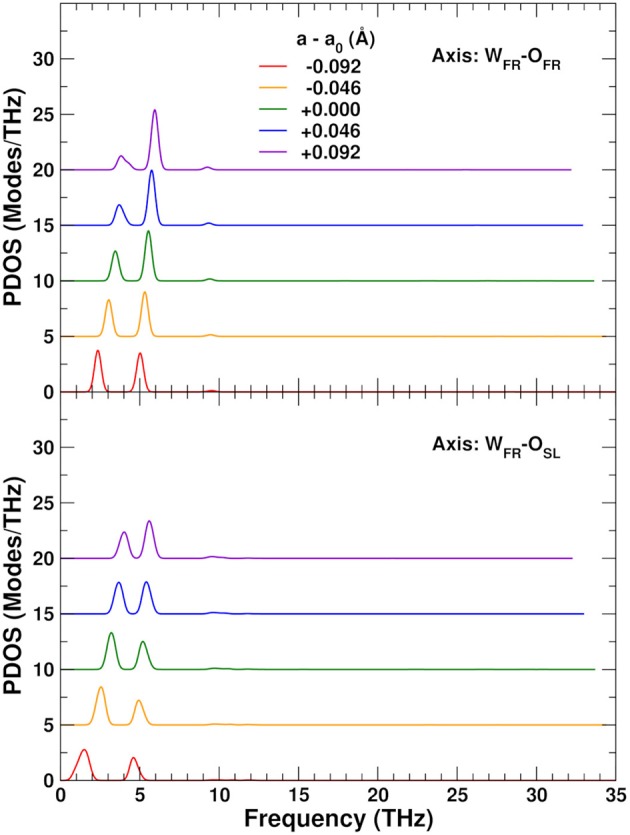
Variation of the ZrW_2_O_8_ phonon density of states (PDOS) projected onto rotational coordinates around the W_FR_–O_FR_ and W_FR_–O_SL_ axes, as a function of lattice constant. The curves for different lattice constants have been vertically shifted for clarity.

To explore the role of the rigid WO_4_ units on the NTE, we have also projected the PDOS onto rotational linear combinations of the transverse in-plane and out-of-plane distortions described above, with axes along the different W–O bonds (Figures 9, 10). These projections eliminate all the internal O–W–O bending contributions seen in the 5–12 THz range of Figure 8, with only features associated with O–Zr–O bending remaining in that range. All these PDOS features show the expected negative Grüneisen parameters γ, and point to the need for a mixed model that is neither purely rigid (Hammonds et al., 1996; Pryde et al., 1996, 1997; Hancock et al., 2004; Tucker et al., 2005, 2007) nor fully distorted (Cao et al., 2002, 2003; Bridges et al., 2014) but one in which the NTE arises from the rigid motion of WO_4_ units and the internal distortion of the ZrO_6_ ones.

### 3.6. Simple NTE model

Based on the results discussed in the previous sections, we are led to formulate a simple anharmonic two-frequency Einstein model to describe the NTE. This model is based on approximations to the different contributions to *A*(*a, T*) and *F*(*a, T*) in Equations (2) and (3). Near equilibrium the internal energy *U*(*a*) can be approximated accurately with a harmonic potential $U\left(a\right)=\left(1/2\right)K_{U}{\left(\Delta a\right)}^{2}$, and Δ*a* ≡ *a* − *a*_0_. The VFE *F*(*a, T*) can be simplified by analyzing the different contributions arising from the PDOS of Figure 7: We have found that the total O-PDOS can be reduced to a very good approximation to just two constant weight poles representing the active regions of the O-PDOS: one for the low frequency (*L*) region below 5.4 THz with a negative Grüneisen coefficient, and a second high frequency (*H*) pole for the modes above 20 THz with a positive Grüneisen coefficient. The intermediate region of the O, Zr, and W PDOS do not contribute to the NTE since they are nearly constant with Δ*a*. The contribution from the *H* pole can be further simplified by noticing that the *H* modes only contribute to *F*_0_(*a*) since at the temperatures relevant here the *H* modes are inactive. We can also take advantage of the linear behavior of the force constants as a function of Δ*a* as discussed in section 2.4: For a given pole ν with effective force constant *k*_ν_, we can write *k*_ν_ = *k*_2ν_ + 6*k*_3ν_Δ*a* (Frenkel and Rehr, 1993; Vila et al., 2007), where *k*_2ν_ and *k*_3ν_ are, respectively, the effective quasi-harmonic and anharmonic force constants. Thus, the frequency ω_ν_ associated with this pole can be approximated as

(10) $$\begin{array}{l} \omega_{\nu }=\sqrt{\frac{k_{\nu }}{\mu_{\nu }}}=\sqrt{\frac{k_{2\nu }+6k_{3\nu }\Delta a}{\mu_{\nu }}}≃\omega_{\nu }^{0}+\omega_{\nu }^{\prime }\Delta a, \end{array}$$

where we define $\omega_{\nu }^{0}=\sqrt{k_{2\nu }/\mu_{\nu }}$, $\omega_{\nu }^{\prime }=3k_{2\nu }/\omega_{\nu }^{0}k_{3\nu }$, and μ_ν_ is the reduced mass associated with this pole. The linear behavior of the pole frequencies around *a*_0_ can be seen in the PDOS of Figure 7, from which the $\omega_{\nu }^{0}$ and $\omega_{\nu }^{\prime }$ parameters can be obtained. The poles weights *w*_*L*_ and *w*_*H*_ are taken at Δ*a* = 0. With these approximations the Helmholtz free energy from Equation (2) can be written as:

(11) $$\begin{array}{l} A\left(a,T\right)=\frac{1}{2}K_{U}{\left(\Delta a\right)}^{2}+\underset{\nu \;=\;L,H}{\sum }F_{0}^{\nu }\left(a\right)+F_{T}^{L}\left(a,T\right), \end{array}$$

where $F_{0}^{\nu }\left(a\right)$ and $F_{T}^{\nu }\left(a,T\right)$ are, respectively, the zero-point and temperature components of *F*(*a, T*) for ν = {*L, H*}. We can now find *a*(*T*) by applying the minimum condition in Equation (1) to the above *A*(*a, T*):

(12) $$\begin{array}{l} 0=\frac{\partial A(a,T)}{\partial a}{|}_{a(T)}= \end{array}$$

(13) $$\begin{array}{l} \;=K_{U}\Delta a+\frac{\hbar }{2}\sum_{\nu =L,H}w_{\nu }{\omega^{\prime }}_{\nu }+\frac{\partial F_{T}^{L}(a,T)}{\partial a}{|}_{a(T)}, \end{array}$$

The last term in this expression can be further simplified by noticing in Figure 4 (bottom) the nearly linear behavior of *F*(*a, T*) around *a*_0_, and expanding $F_{T}^{L}\left(a,T\right)$ to first order in *a*. After this, we can calculate *a*(*T*) simply as:

(14) $$\begin{array}{l} a\left(T\right)≃a_{0}-\frac{\hbar }{K_{U}}\underset{\nu =L,H}{\sum }w_{\nu }\omega_{\nu }^{\prime }+\frac{\hbar w_{L}\omega_{L}^{\prime }}{K_{U}\left[1-e^{\hbar \omega_{L}^{0}/k_{B}T}\right]}, \end{array}$$

using the internal energy minimum and constant *a*_0_ = 9.246 Å and *K*_*U*_ = 85.82 eV/Å^2^, respectively, weights *w*_*L*_ = 21.1 and *w*_*H*_ = 30.0, harmonic frequency $\omega_{L}^{0}=20.31$ THz, and effective anharmonic constants $\omega_{L}^{\prime }=81.90$ THz/Å and $\omega_{H}^{\prime }=-104.39$ THz/Å. Our results are presented in Figure 5; they clearly show that this simplified two-pole Einstein model yields a quantitative description of the observed NTE. Of these contributions the *L* pole dominates but overestimates the NTE, while the *H* pole provides a 30% correction.

## 4. Conclusions

We have performed calculations of the thermal expansion of ZrW_2_O_8_ using an efficient approach for obtaining the variation of the dynamical matrix as a function of the lattice constant. The anomalous NTE arises almost exclusively from the transverse contributions of the O-atoms, as demonstrated by a partition of the vibrational free energy over the O, W, and Zr sites. This behavior results from the interplay between the normal positive Grüneisen parameter and hence PTE of the O-projected high frequency modes, and the anomalous negative-Grüneisen parameter driving the NTE from the low frequency modes. The low-frequency modes overestimate the effect of NTE, while the high-frequency modes provide a 30% correction. These low frequency modes are associated with distortions at the O sites transverse to the W–O or W–O–Zr bonds. More precisely, the rotational linear combination of these transverse distortions results in nearly rigid rotations of the WO_4_ tetrahedra and internal distortions of the ZrO_6_ octahedra. The mixed character of the low frequency modes is consistent with the variety of models previously proposed to account for the NTE. We have simplified this complex behavior by proposing a two-frequency anharmonic Einstein model of the O-PDOS which captures both the dominant negative- and weak positive-contributions to thermal expansion.

## Author contributions

FV co-developed the theory, was the main developer of the specialized software used in the analysis, and performed most of the simulations. SH contributed to the simulations and helped in verifying the soundness of the software. JR co-developed the theory presented here.

### Conflict of interest statement

The authors declare that the research was conducted in the absence of any commercial or financial relationships that could be construed as a potential conflict of interest.

## Supplementary material

Supplementary Data Sheet 1 Numerical data for all plots in this work.
